# *Ex Vivo* Human Adipose Tissue Derived Mesenchymal Stromal Cells (ASC) Are a Heterogeneous Population That Demonstrate Rapid Culture-Induced Changes

**DOI:** 10.3389/fphar.2019.01695

**Published:** 2020-02-20

**Authors:** Anna E. S. Brooks, Megan Iminitoff, Eloise Williams, Tanvi Damani, Victoria Jackson-Patel, Vicky Fan, Joanna James, P. Rod Dunbar, Vaughan Feisst, Hilary M. Sheppard

**Affiliations:** ^1^School of Biological Sciences, University of Auckland, Auckland, New Zealand; ^2^Maurice Wilkins Centre, University of Auckland, Auckland, New Zealand; ^3^Department of Obstetrics and Gynecology, University of Auckland, Auckland, New Zealand

**Keywords:** human adipose tissue-derived mesenchymal stem/stromal cells, adipose-derived stem cell, mesenchymal stem cells, paracrine effect, heterogeneity, immunomagnetic bead sorting, flow cytometry, stromal vascular fraction

## Abstract

Human adipose-derived mesenchymal stromal cells (ASC) are showing clinical promise for the treatment of a range of inflammatory and degenerative conditions. These lipoaspirate-derived cells are part of the abundant and accessible source of heterogeneous stromal vascular fraction (SVF). They are typically isolated and expanded from the SVF *via* adherent cell culture for at least 2 weeks and as such represent a relatively undefined population of cells. We isolated *ex vivo* ASC directly from lipoaspirate using a cocktail of antibodies combined with immunomagnetic bead sorting. This method allowed for the rapid enrichment of a defined and untouched *ex vivo* ASC population (referred to as MACS-derived ASC) that were then compared to culture-derived ASC. This comparison found that MACS-derived ASC contain a greater proportion of cells with activity in *in vitro* differentiation assays. There were also significant differences in the secretion levels of some key paracrine molecules. Moreover, when the MACS-derived ASC were subjected to adherent tissue culture, rapid changes in gene expression were observed. This indicates that culturing cells may alter the clinical utility of these cells. Although MACS-derived ASC are more defined compared to culture-derived ASC, further investigations using a comprehensive multicolor flow cytometry panel revealed that this cell population is more heterogeneous than previously appreciated. Additional studies are therefore required to more precisely delineate phenotypically distinct ASC subsets with the most therapeutic potential. This research highlights the disparity between *ex vivo* MACS-derived and culture-derived ASC and the need for further characterization.

## Introduction

Human adipose tissue derived stromal cells (ASC) are currently being tested as cell-based therapies against a wide range of diseases and conditions in numerous clinical trials (Bateman et al., 2018) including wound healing, (Bertozzi et al., 2017) cardiovascular disease (Ma et al., 2017) and cartilage regeneration (Pak et al., 2018). ASC are isolated from adipose tissue and are therefore an abundant source of stromal cells that can be accessed with relative ease using the minimally invasive procedure of liposuction. ASC are often referred to as adipose tissue derived “stem” cells (ASC or AdMSC) or mesenchymal stem cells (MSC), inferring their suitability for a wide range of regenerative applications. However, the cellular diversity of this population, referred to hereafter as adipose tissue derived mesenchymal stromal cells (ASC), and their true therapeutic potential remains unclear. They are defined in part as being able to differentiate into fat, bone, and cartilage lineages *in vitro* (Bourin et al., 2013). They are also reported to function as bioreactors producing molecules that promote healing and inhibit over activity of the immune system (Ma et al., 2014). Although their exact therapeutic mode of action *in vivo* is unclear (Robey, 2017) increasing evidence points to mesenchymal cells exerting a paracrine effect (Zwolanek et al., 2017; Caplan, 2019) rather than cell replacement.

To isolate ASC, the by-product of liposuction, termed the lipoaspirate, is digested with collagenase and centrifuged resulting in a cell pellet known as the stromal vascular fraction (SVF). This is a heterogeneous mix of cells including ASC, preadipocytes, endothelial cells, and immune cell subsets. A widely used method to enrich for ASC involves culturing the SVF cell pellet *in vitro*. In 2006, the International Society for Cell and Gene Therapy (ISCT), formerly known as the International Society for Cellular Therapy, proposed a minimum set of guidelines to define cells isolated from tissue (Dominici et al., 2006). These included (1) that morphologically the cells are plastic adherent and fibroblastic (2) that they express the cell surface markers CD73, CD90, and CD105, and do not express haematopoietic and endothelial antigens (CD14 or CD11b, CD19 or CD79α, CD34, CD45, HLA-DR) and (3) that they display “trilineage potential” in that they able to differentiate into adipocytes, osteoblasts, and chondrocytes *in vitro*. This definition was later extended in a position statement released by the ISCT and International Federation for Adipose Therapeutics and Science (IFATS) in 2013 to include the preculture or *ex vivo* criteria to define ASC within SVF. In this position statement, the phenotypic characteristics of ASC isolated from SVF were further refined to include CD34 as a positive marker, a key difference between culture-isolated and *ex vivo* ASC (Bourin et al., 2013). Collectively, these criteria have provided a useful common ground in the mesenchymal field. Nevertheless there is now increased awareness that these definitions are no longer an up-to-date reflection of the knowledge that is rapidly accumulating. In addition, *in vitro* differentiation assays, which require cocktails of chemical cues, do not necessarily mimic the *in vivo* environment, nor demonstrate an accurate reflection of the *in vivo* activity of the cell (Locke et al., 2011; Robey, 2017). Furthermore, these defined cell surface markers are also expressed by cultured fibroblastic cells from a variety of tissue sources. It is also becoming increasingly apparent that the ASC fraction itself is heterogeneous (Merrick et al., 2019). Therefore further studies are required to identify ASC defining markers to enable the enrichment of a more defined population of cells (da Silva Meirelles et al., 2006; Crisan et al., 2008; Nielsen et al., 2016).

The plastic-adherent culturing method used to isolate a “pure” population of ASC from the SVF typically requires a minimum of 2–3 weeks in culture and even then the population can be far from homogenous (Ho et al., 2008; Baer et al., 2013). However, it should be noted that currently there is a lack of consistency or standardisation regarding the preparation of ASC for use in the clinic. Increased time in culture may increase apparent homogeneity (Mitchell et al., 2006), however culture duration could affect clinical utility and lead to increased production times, costs, and regulatory hurdles associated with getting a product to the clinic. In addition, the incidence of genetic abnormalities tends to increase with time in culture (Neri et al., 2013), therefore minimizing passage number may improve the safety profile of cells. Finally, increased passage number has been reported to result in decreased potency (Wall et al., 2007; Park et al., 2011; Lo Surdo et al., 2013). To assess what effect cell culture may have at the functional and molecular level we sought to compare culture-derived ASC with an uncultured *ex vivo* population with a defined cell-surface phenotype based on the ISCT/IFATs recommendation (Bourin et al., 2013). To this end we report here on the use of an immunomagnetic bead approach to rapidly enrich a defined and untouched population of *ex vivo* ASC from the SVF, hereafter referred to as MACS-derived ASC. To our knowledge, a side by side comparison of *ex vivo* and culture-derived ASC has not been performed previously. We hypothesised that this comparison would be important to help to elucidate the clinical utility of these two cell populations.

We found that MACS-derived ASC contain a greater proportion of cells with activity in *in vitro* differentiation assays compared to culture-derived ASC and that they exhibit an altered profile of secreted proteins. These differences may reflect the undefined nature of culture-derived ASC expanded from SVF. In addition, we show that culturing *ex vivo* MACS-derived ASC rapidly alters their gene expression profile in ways that may affect their clinical utility. This suggests that methods that enrich for a defined population of uncultured ASC may be beneficial to clinical utility in some settings. MACS-derived ASC appear homogenous based on their cell surface phenotype (according to the ISCT/IFATS definition (Bourin et al., 2013). However, using a comprehensive multicolor flow cytometry panel, here we further demonstrate that this population of cells is more heterogeneous than previously reported (Bourin et al., 2013) and is variable between donors. This highlights the need to further characterize the functionality of defined subpopulations of ASC to improve reproducibility of results in this field. Our research highlights the disparity between *ex vivo* MACS-derived and culture-derived ASC populations and the need for further characterization.

## Materials and Methods

### Processing Lipoaspirate

Lipoaspirate was obtained from informed healthy, nonobese, female donors undergoing elective liposuction with protocols approved by the Northern A Health and Disability Ethics Committee (approval number NTX/07/02/003). One litre of lipoaspirate was washed twice with an equal volume of phosphate-buffered saline (PBS) and digested with 0.15% Collagenase type I (Life Technologies) in PBS for 60 min at 37°C with occasional mixing. Cells were pelleted by centrifugation at 690 g for 10 min at room temperature resulting in the SVF. The pellet was resuspended in 50 ml prewarmed ASC medium (Dulbecco’s modified eagle media/Ham’s F12 nutrient mixture (DMEM F-12; Life Technologies) supplemented with 10% Fetal Bovine Serum (FBS) (Life Technologies), 1% Penicillin-Streptomycin 10,000 U/ml (Invitrogen), and 1 × GlutaMAX (Invitrogen) and passed through a 100-μm Falcon™ cell strainer (BD). SVF was pelleted again and resuspended in 50% ASC media and 50% freezing media (FBS plus 20% DMSO (Sigma-Aldrich) and cryopreserved in liquid nitrogen. From eight donors the average SVF yield from 1 L of lipoaspirate was 3 × 10^8^ cells (data not shown).

### Culturing SVF to Isolate Culture-Derived ASC

Frozen SVF suspensions were thawed and for each donor vials were split to allocate half for MACS sorting and half for plastic adherent culture. Both isolation methods were conducted in parallel and cells from each donor were cultured separately. On average 5 × 10^6^ cells were plated into a Falcon™ T75 tissue culture flask in ASC medium (see above). When cells reached 90% confluency they were detached from the flask using trypLE (ThermoFisher) and passaged at a 1 in 2 dilution, typically once a week, for 28 days (four passages). Cell purity was assessed by flow cytometry as described below for MACS sorted cells. Morphology images were taken using a Leica DMI3000 B Inverted Microscope equipped with a Leica DFC290 camera and Leica Application Suite (LAS) software.

### Flow Cytometry

All FACS cell sorting and analyses were performed on a BD SORP FACS Aria II equipped with four lasers (see **Supplementary Table 1**). Voltration experiments were performed to optimize PMT Voltages across all detectors using unstained lymphocytes and Mid Intensity beads (BioLegend). This process involved stepwise increments in voltage gain to determine the minimal voltage required to ensure that dim signals were above electronic noise and within the linear detection range. These optimal settings were then saved as “application settings” and were used for all experiments to ensure consistency between experiments. All flow cytometry reagents were titrated to determine optimal dose and panels developed to minimize spectral overlap. For all experiments single-stained controls were prepared with BD CompBeads Plus, except for CD34 and CD90 where single cell controls were prepared instead. Compensation was done using the automated wizard in BD FACSDiva. All data analyses were performed using FlowJo V10.2 (BD Biosciences, San Jose, CA).

### MACS Sorting Cells to Isolate MACS-Derived ASC

Frozen SVF suspensions were thawed and for each donor vials were split to allocate half for MACS sorting and half for plastic adherent culture. ASC were enriched using MACS™ anti-FITC microbeads (Miltenyi) according to the manufacturer’s instructions with an antibody cocktail consisting of 2.5 µl of each of the following antihuman FITC-conjugated antibodies: CD31 (clone MW59), CD45 (clone HI30), CD146a (PIH12), and CD235a (clone H1246) (all from BioLegend). In brief, the SVF single cell suspension was pelleted and resuspended in 100 μl MACS buffer per 10^7^ cells and incubated with the antibody cocktail on ice for 10 min protected from light. Cells were washed twice with MACS buffer and resuspended in 90 μl of buffer per 10^7^ cells. Cells were then incubated with 10 μl of anti-FITC MACS microbeads (Miltenyi) per 10^7^ cells for 15 min at 4°C and washed with MACS buffer. These were resuspended in 500 μl of buffer and applied to a precooled LS column (Miltenyi). Post sort purity was assessed by flow cytometry using CD73, CD90, CD31, CD45, CD34, and CD146 antibodies (see **Supplementary Table 2**).

### Adipogenic Differentiation Assays

Adipogenic staining was performed as described previously (Eom et al., 2018). In brief cells were plated in a 96-well plate in 200 μl ASC media (DMEM/F12 media (Life Technologies) supplemented with 10% FBS, 1% GlutaMax (Life Technologies, Auckland) and 1% penicillin/streptomycin (Life Technologies) and cultured at 37°C, 5% CO_2_. On day four, 100 μl of media was replaced with adipogenic differentiation media (ASC media with 1 µM dexamethasome, 10 µM insulin, 0.5 mM 3-isobutyl-1-methylxanthine (IBMX), and 200 µM indomethacin (all from Sigma Aldrich, Auckland) and standard ASC media was added to control wells. Half media changes were performed every 3 days until day 14. Cells were then subjected to immunocytochemistry using a 1:200 dilution of rabbit antihuman FABP4 polyclonal antibody (Cat #10004944, Cayman Chemicals) and then incubated with a 1:200 dilution Alexa Fluor^®^ 488 conjugated goat antirabbit IgG secondary antibody (Cat # A11008, Molecular Probes^®^) and 1:2,000 diluted DAPI (Cat# D3571, Molecular Probes^®^). Fluorescent images were taken using the ImageXpress Micro XLS high content screening system (Molecular Devices™). Nine images were taken per well at 10 × magnification and quantitative data was generated using the MetaXpress v 5.3.0.1 (Molecular Devices™) software.

### Osteogenic Differentiation Assay

ASC were seeded into a 96-well plate in standard ASC media. The following day half of the media was replaced with StemPro^®^ osteogenic differentiation medium in experimental wells and with standard ASC medium in control wells. Half media changes were performed with osteogenic media every three days until day 21. Cells were then fixed with 4% formaldehyde for 30 min at room temperature, washed twice with water, then incubated with 2% Alizarin Red 5 min at room temperature. Cells were washed with water, dried and imaged (12 images per sample) using a Leica DMI3000 B Inverted Microscope equipped with a Leica DFC290 camera. Alizarin Red stain was quantified using Image J software.

### Microarrays

Cells were FACS sorted on a BD SORP FACS Aria II using the same antibody cocktail as described for the MACS sort, and post sort analysis was as above. Cells were washed once in ice-cold PBS and total RNA was purified using the miRVANA kit (Ambion). RNA integrity was assessed using a Bioanalyser (Agilent). 100 ng of RNA were reverse transcribed and labeled using the Genechip 3’ IVT Express kit and hybridised to Primeview Arrays (Affymetrix) according to manufacturer’s protocols. Fluorescent signals were recorded by an Affymetrix scanner 3000 using Gene Chip Operating Software. The Affymetrix^®^ Expression Console™ Software was used to carry out quality control analysis. Affymetrix^®^ Transcriptome Analysis Console (TAC) 3.0 was used to determine genes that were differentially expressed between different conditions (day 0, day 3 and day 28). Only those genes that showed an ANOVA p-value of less than 0.05 and a fold difference between > 2 and < −2 were considered as differentially expressed between the conditions. The p-value and fold differences were an average obtained from three different donors. Data was preprocessed, normalized, and summarized using the RMA (Irizarry et al., 2003) function from the R software package affy (Gautier et al., 2004). A dendrogram was used to show a pictorial representation of the relationship between the samples. The Euclidean distance between the normalized samples were calculated, then clustered together using the Ward’s method. The dendrogram showing the grouping of the clustered samples was then plotted using the ape (Paradis and Schliep, 2018) package. A list of transcripts with a fold change of greater than, or equal to 10 was obtained using the Affymetrix/Thermofisher Transcriptome Analysis Software. A heatmap of these transcripts were plotted using the heatmap.2 function from the gplots (Warnes et al., 2016) package. Microarray data has been deposited in the Gene Expression Omnibus (GEO) database and can be accessed *via* accession no. GSE136633.

### Quantitative Real Time (RT)-PCR

Quantitative real-time (RT)-PCR was performed as described previously (Sheppard et al., 2016). In brief total RNA was isolated from all samples using a miRVANA kit (Ambion). First-strand cDNA was synthesized for all samples using random hexamer primers and SuperScript III reverse transcriptase (Invitrogen). Quantitative RT-PCR was carried out on a 7900HT Real-Time PCR System (Applied Biosystems) using TaqMan^®^ FAST Universal PCR Master Mix (Roche), gene specific TaqMan^®^ probes and between 2 and 10 ng cDNA per reaction. PCR cycling parameters were 20 s at 95˚C and then 40 cycles of 1 s at 95˚C followed by 20 s at 60˚C. Results were normalized against two housekeeper genes (B2M and HPRT1).

### Quantification of Paracrine Factors

MILLIPLEX^®^ MAP kits (EMD Millipore, Merck) were used to quantify key paracrine factors present in the conditioned media of “MACS” or “cultured” isolated cells. Cells isolated by MACS were immediately plated postsort into 1 ml ASC medium in a standard 24-well plate at an equal density to ASC that had been cultured from SVF for at least 2 weeks. Four days later supernatants were removed, centrifuged for 5 min at 360 g at 4°C to pellet cell debris and stored at −20°C until analyzed. At this time, fresh media was added to the cells for further 3 days, before being and harvested again in the same manner. Three Human Cytokine/Chemokine Magnetic Bead Panels were used to quantify cytokines in the supernatents (1) Cat# HCYTOMAG-60K was used to detect IL-6, Il-8, VEGF, and IFNγ, (2) Cat# HIGFMAG-52K was used to detect IGF I and II, and (3) Cat# TGFBMAG-64K-03 was used to detect TGFβ 1 and 2. Samples were run in duplicate on a MAGPIX^®^ analyser and results were obtained using Luminex xPONENT software.

### Multicolor Flow Cytometry Analysis

For phenotypic analyses, frozen cell samples were thawed, washed, and resuspended in ASC medium and incubated at 37°C/5% CO_2_ for 1 hour prior to staining. Cells were stained using an antibody cocktail containing CD26, Podoplanin, CD271, CD144, CD105, CD90, CD36, FAP, CD34, CD73, CD31, HLADR, CD45, CD146, CD141, CD73, CD31, CD45, and CD34 (see **Supplementary Table S2**). Following a 30-min incubation on ice, cells were washed twice in 1 ml of staining buffer (PBS + 1% human serum). Cells were resuspended in buffer and DAPI (1:5,000) was added to exclude dead cells immediately prior to data acquisition on a BD SORP FACS Aria II. Data in **Figure 5A** was analyzed using FlowJo V10.2 (BD Biosciences, San Jose, CA) and in **Figures 5B, C** using viSNE (Amir el et al., 2013) and FlowSOM (Van Gassen et al., 2015) in Cytobank (www.cytobank.org/). viSNE was run using equal sampling (204,977 cells) of the pregated CD90+CD73+CD34+ population to identify heterogeneity within the populations and also to allow for donor comparisons. FlowSOM was subsequently run to identify clusters by hierarchical clustering. Four clusters (tabulated in **Figure 5C**) were identified using two dimensional gating and subsequently displayed as overlays on the viSNE plots.

### Statistical Analysis

Unless otherwise stated, statistical analysis was performed using Microsoft Excel software. Significance was assessed using two-tailed type 1 t-tests with p values < 0.05 being considered significant. * denotes a p value < 0.05, ** denotes a p value < 0.01, and *** denotes a p value < 0.001.

## Results

### MACS Enrichment of a Defined Population of ASC From SVF

**Figure 1:**
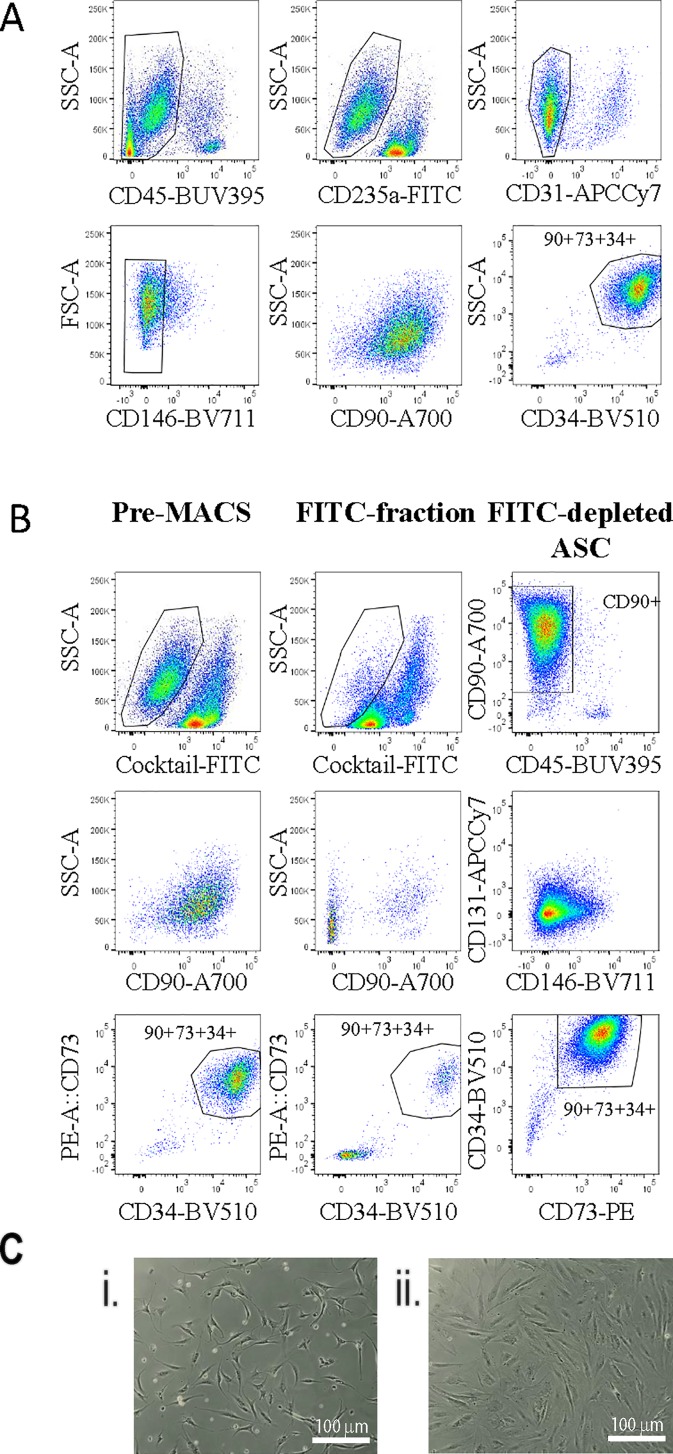
Untouched adipose-derived stem cell (ASC) can be enriched from the stromal vascular fraction (SVF) using an antibody cocktail to deplete contaminating cell populations. A flow cytometry panel was used to demonstrate exclusion gating of CD45, CD31, CD235a, and CD146 cells enriched for CD90+CD73+CD34+ ASC cells within the SVF **(A)**. Therefore, a FITC antibody cocktail containing anti-CD45, -CD31, -CD235a, and -CD146 and anti-FITC microbeads was used to enrich ASC from SVF single cell digests using the “MACS” protocol. **(B)** Flow cytometry was used to assess the enrichment strategy: (1) presort, (2) the FITC+ fraction, and (3) the FITC depleted fraction with a panel of markers including CD73-PE, CD90-A700, CD31-APC-Cy7, CD45-BUV395, CD34-BV510, and CD146-BV711. Data shown are representative of four biological replicates. **(C)** The morphology of MACS-derived four days post sort (i) or culture-derived ASC 48 h post passage four (28 days) (ii) was assessed. This image is representative of at least four donors.

Our previous analysis of the SVF from human adipose tissue using multicolor flow cytometry gave us a good understanding of the various cell types present in this tissue (Feisst et al., 2014). Based on this earlier work we used a flow cytometry panel to demonstrate that the ASC population, positive for CD90, CD73, and CD34, could be enriched by excluding all populations positive for CD45 (haemopoietic), CD235a (red blood cells), CD31 (endothelial), and CD146 (pericytic) (**Figure 1A**). Therefore, we sought to use a cocktail of FITC-labeled antibodies (CD31, CD45, CD146, and CD235a) and anti-FITC labeled magnetic MACS™ beads to enable enrichment of an “untouched” ASC cell population from the SVF using immunomagnetic column-based sorting. Samples labeled with the anti-FITC cocktail and anti-FITC beads were passed over a magnetic column, to allow the enrichment of untouched ASC cells to flow through. The presort, column-retained (FITC-positive), and enriched/flow through fractions were analyzed by flow cytometry (**Figure 1B**). Flow cytometry analyses indicated that post sort purity of the enriched fraction was >97% for CD34+, CD73+, and CD90+ (see representative data from one donor in **Figure 1B**). Cell morphology of cells sorted using the immunomagnetic bead protocol (herein referred to as MACS-derived cells) was comparable to stromal cells isolated from the same donors in parallel using the standard method of plastic adherence and 28 days in culture (herein referred to as culture-derived cells) (**Figure 1C**).

**Figure 2:**
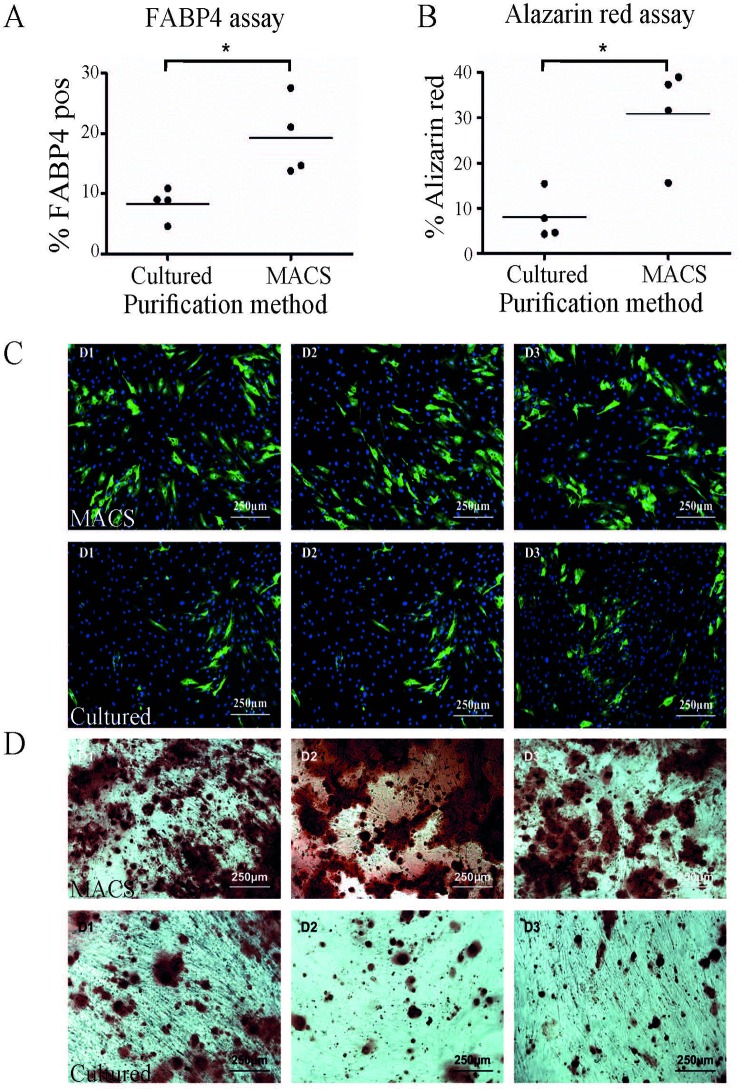
A greater proportion of MACS-derived adipose-derived stem cell (ASC) exhibit activity in *in vitro* differentiation assays compared to culture-derived ASC. **(A)** MACS-derived and culture-derived ASC were subjected to an *in vitro* adipogenic differentiation assay. The graph represents the percentage of cells which stained positive for FABP4 expression after quantification. Panel **(C)** shows representative images from the adipogenic differentiation assay for three donors (D1–D3) where FABP4 positive cells are stained green and nuclei are stain blue. The top row contains images of MACS-derived ASC and the bottom row contains images from culture-derived ASC. The graph in **(B)** represents the percentage of cells which stained positive for alizarin red after 3 weeks of culture in commercial osteogeneic differentiation media. Panel **(D)** shows representative images from the alizarin red assay for three donors (D1–D3). The top row contains images of MACS-derived ASC and the bottom row contains images from culture-derived ASC. * denotes a p value of < 0.05.

One of the most common methods to enrich for ASC is by plastic adherence, followed by cell expansion and passaging. We have previously demonstrated that 28 days was sufficient to achieve homogeneity of ASC based on CD90, CD73, and CD34 (Feisst et al., 2014) expression and are consistent with the IFATS/ISCT definition for ASC (Bourin et al., 2013). In addition three to four passages is generally accepted as a pure population (Braun et al., 2013) and is comparable to standard methods used to enrich for ASC. We therefore compared MACS-derived cells to culture-derived cells that had been cultured for 28 days. Unsurprisingly, this results in higher cell yields compared to MACS-derived cells. The cultured-derived method generated at least six times more cells (19.12 × 10^6^, st. dev +/− 4.0 × 10^6^) after 28 days in culture compared to the MACS enrichment process (2.9 × 10^6^, st. dev +/− 0.62 × 10^6^), n = 4). This method infers that large cell numbers are more clinically relevant; however, it is also likely that this period in culture will influence the functional capacity of these cells. In addition, a loss of ASC differentiation potential over time in culture has also been reported (Wall et al., 2007; Park et al., 2011; Lo Surdo et al., 2013). However, MACS enriched cells represent a more defined population that may be more potent in some settings. Therefore, we hypothesised that the MACS-derived ASC would perform better in *in vitro* differentiation assays compared to culture-derived ASC, although we acknowledge that these assays do not necessarily reflect *in vivo* activity. When subjected to a quantitative adipogenic differentiation assay assessing FABP4 expression by immunohistochemistry, a significantly greater proportion of MACS-derived cells expressed FABP4 in comparison to culture-derived ASC isolated from the same donor (**Figures 2A, C**). When subjected to a semi-quantitative osteogenic differentiation assay, using alizarin red staining as a marker of calcium rich deposits, we also consistently observed significantly higher levels of alizarin red staining in the MACS-derived ASC cells in comparison to culture-derived ASC isolated from the same donor (**Figures 2B, D**).

Next, we sought to examine the molecular changes that might occur when enriched *ex vivo* ASC are cultured *in vitro*. In this experiment ASC were enriched by flow assisted cell sorting (FACS) using the same antibody cocktail as was used for the MACS bead enrichment described above. The sorted ASC were then grown on plastic in standard tissue culture conditions for 0, 3, or 28 days, after which total RNA was isolated. This approach meant that the same defined population of cells was being assessed at each time point with the major experimental variable being time in culture. These time-points were chosen to examine gene expression in uncultured (day 0), minimally cultured (day 3), and extensively cultured *ex vivo* FACS-derived ASC (day 28). The 28-day time-point was chosen to match the time point used to enrich for culture-derived ASC from SVF. RNA was subjected to microarray analysis using Affymetrix PrimeView arrays. Quality controlled and robust microarray average (RMA) normalized data was further analyzed using Affymetrix transcriptome analysis console software to identify any genes differentially expressed between the treatment groups.

**Figure 3:**
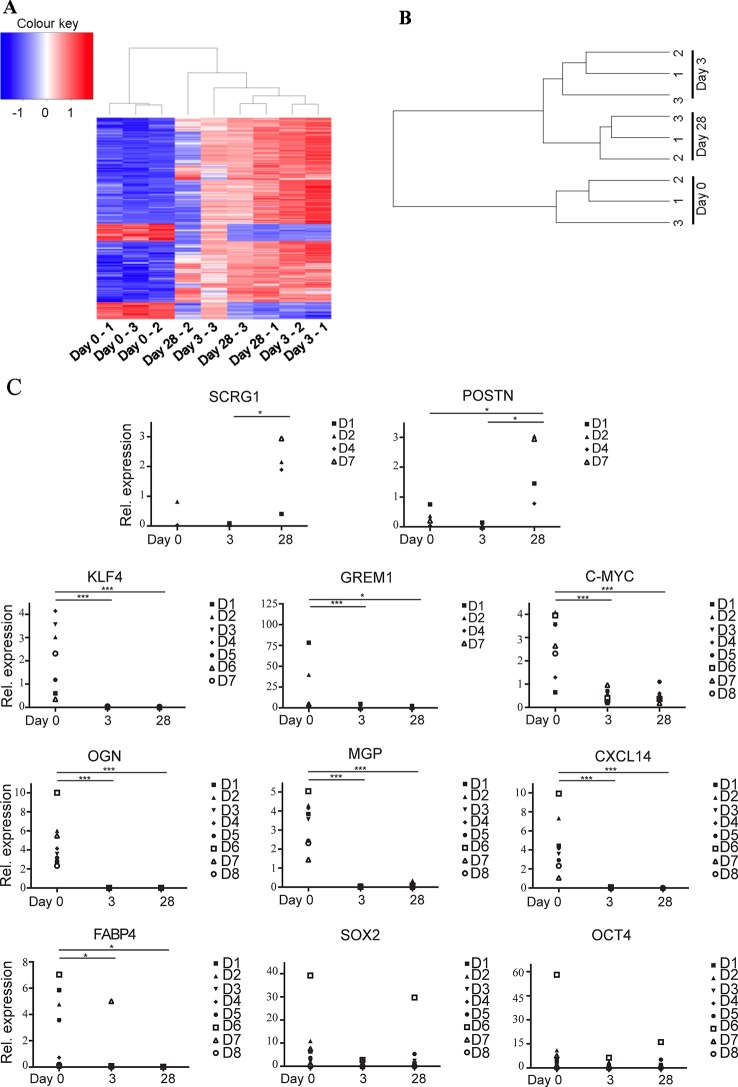
*Ex vivo* MACS-derived adipose-derived stem cell (ASC) exhibit rapid and marked changes in gene expression when subjected to standard tissue culture conditions. MACS-derived ASC were subjected to standard tissue-culture conditions and microarray analysis was performed at day 0, day 3, or day 28 post sort. **(A)** A heatmap was generated using a list of all transcripts which exhibited a fold change >10 when microarray data was analyzed using the Affymetrix Transcript Analysis Console software. Blue represents downregulated genes and red upregulated genes from three donor samples (1–3) at day 0, day 3, or day 28 post sort. **(B)** Unsupervised clustering was performed using all transcripts detected in the microarray data when processed using robust microarray average (RMA). This was then plotted as a dendogram to pictorially represent the relationship between the three donor samples (1–3) at day 0, day 3, or day 28 post sort. **(C)** Real-time PCR was used to validate a subset of the microarray data (target genes SCRG1, POSTN, KLF4, GREM1, C-MYC, OGN, MGP, CXCL14, FABP4, SOX2, and OCT4 as indicated) in at least four, and a maximum of eight subsequent donors (D1–D8). * denotes a p value of < 0.05, *** denotes a p value of < 0.001.

49,372 genes were interrogated using the microarray platform. When comparing freshly sorted “day 0” cells to cells cultured for 3 days (“day 3”) from three donors 1,122 genes were downregulated > twofold (with an ANOVA p value < 0.05) and 13,659 genes were upregulated > twofold. When comparing day 0 to day 28 cells these numbers change to 3,018 genes downregulated and 6,358 genes upregulated. A comparison of differential gene expression between day 3 and day 28 cells indicated that only 197 genes are downregulated and 853 genes are upregulated (see **Supplementary Table S3** for genes upregulated and downregulated > tenfold comparing day 0 versus day 3, day 3 versus day 28, and day 0 versus day 28). This data indicates that global gene expression changes are most striking during the first three days in tissue culture conditions and changes thereafter are more modest. A heat map generated using a list of all transcripts which exhibited a fold change >10 clusters uncultured cells together and cultured cells as a separate group, regardless of time spent in culture (**Figure 3A**). This is further demonstrated by unsupervised hierarchical clustering based on all gene expression changes which again groups all cultured cells together as a separate group from the uncultured cells, regardless of time spent in culture (**Figure 3B**).

Lineage-specific genes were among the top most significantly downregulated genes following a 28-day culture period (day 0 versus day 28). This included genes typically implicated in the terminal differentiation of MSCs following commitment to the chondrogenic, osteogenic, and adipogenic fates including matrix Gla protein (MGP, > 48-fold), osteoglycin (OGN, > 330-fold down) and fatty acid binding protein 4 (FABP4, > 790-fold) respectively. KLF4, a transcription factor present in pluripotent stem cells was also downregulated > eightfold, although other pluripotent factors such as c-MYC, SOX2, OCT4, and NANOG were not found to be differentially expressed. Many immune response related genes were also downregulated between day 0 and day 28 including chemokines (CXCL14 (> 800-fold) CXCL12 (> 19-fold), CXCL3 (> 18-fold) and CXCL2 (> 29-fold)) and HLA-DRa (> 28-fold), CD14 (43-fold), and CD54 (> 17-fold).

Functional annotation analysis using the DAVID tool (Huang da et al., 2009) on genes downregulated > twofold between days 3 and 28 ranks positive regulation of cell proliferation followed by negative regulation of apoptosis as the pathways most affected by time in culture (p values of > 0.001). This suggests that over time proliferation decreases and apoptosis increases. Numerous ECM, adhesion, cytoskeletal, and matrix remodelling proteins were among the genes upregulated following a 28-day culture period (**Supplementary Table S3**). DAVID analysis on genes upregulated > twofold between days 3 and 28 ranks extracellular matrix reorganization, cell adhesion, collagen fibril organization, and regulation of cell shape as the top four pathways most affected by time in culture (p values of > 0.001).

Real-time PCR was used to validate some of the differentially expressed genes identified by the microarray analysis using ASC isolated from up to eight subsequent donors (**Figure 3C**). MACS-derived ASC (day 0) were compared to cultured expanded MACS-derived ASC grown in standard tissue culture conditions for 3 or 28 days. Significant downregulation of KLF4, FABP4, OGN, MGP, and CXCL14 and significant upregulation of SCRG1 and POSTN was confirmed. We also assessed the expression of the pluripotency associated genes c-MYC, SOX2, and OCT4 that had not been identified as differentially expressed by the microarray data. c-MYC was significantly, albeit modestly, reduced in expression whilst no differential expression was observed for SOX2 or OCT4. ASC from donors 4–8 were all sorted from the SVF *via* MACS, whereas ASC from donors 1, 2, and 3 were sorted *via* FACS. Comparison of the qPCR data derived from MACS or FACS sorted cells was performed using one-way ANOVA and indicated that there were no statistical differences between these two sorting methods (data not shown).

**Figure 4:**
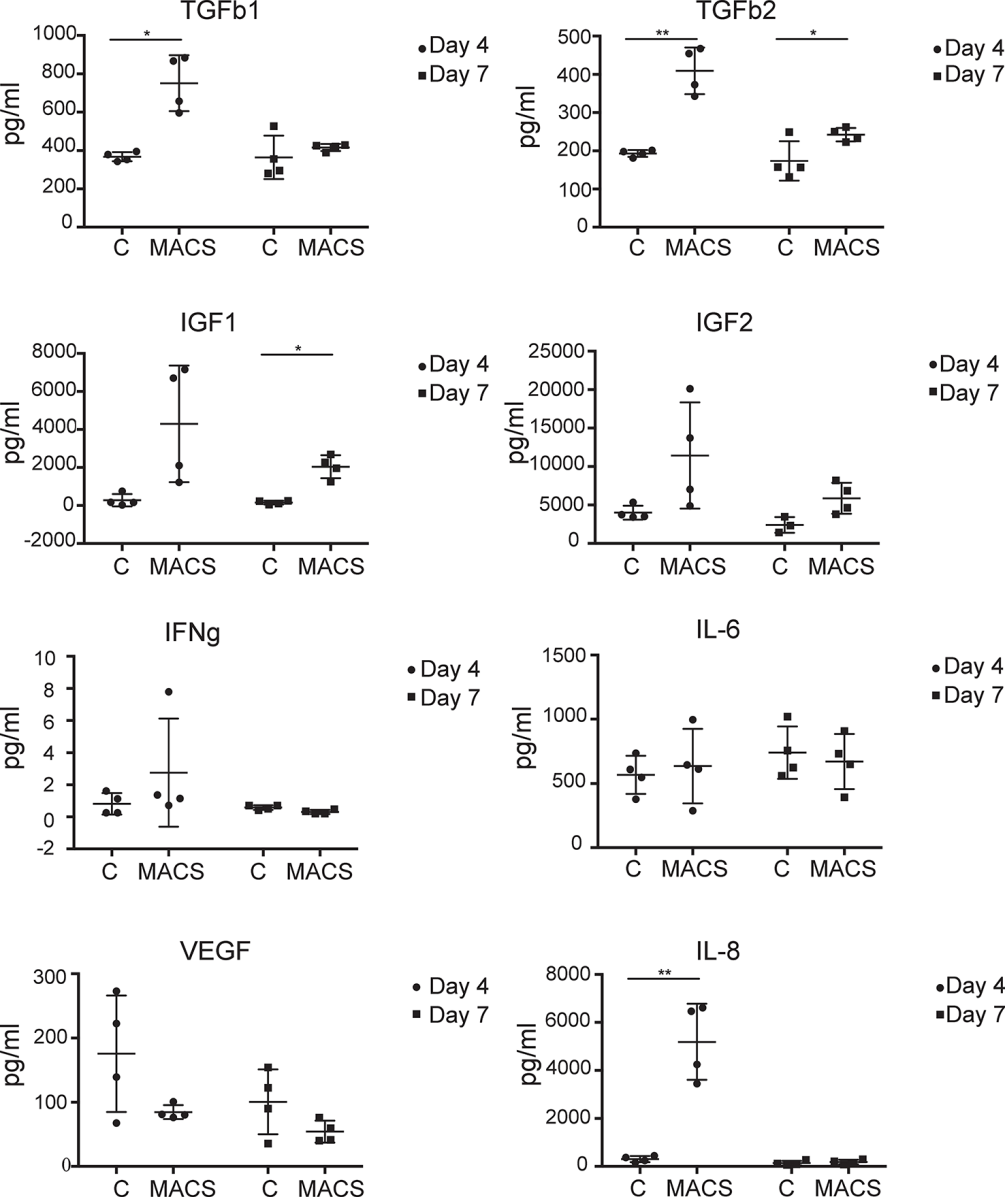
MACS-derived adipose-derived stem cell (ASC) differentially secrete key paracrine-effect proteins compared to culture-derived ASC. Milliplex^®^ multiplexed assay panels were used to assess the expression levels of key secreted proteins thought to be involved in the paracrine effect of ASC. Each panel represents the expression level of a specific protein (TGFb1, TGFb2, IGF1, IGF2, IFNg, IL-6, and VEGF as indicated) present in the media supernatant from culture-derived ASC (C) or MACS-derived (MACS), in both cases 4 days or 4–7 days (day 7) post plating at equal densities. Data derived from four donors. * denotes a p value of < 0.05, ** denotes a p value of < 0.01.

ASC are thought to exert beneficial clinical immune-modulatory effects in part *via* a paracrine mechanism (Kilroy et al., 2007; Ma et al., 2014). Therefore, we were interested to assess if key secreted molecules were differentially present in conditioned media taken from MACS-derived ASC compared to culture-derived ASC. The microarray data, which focused only on MACS-derived ASC, suggested that some secreted chemokines and growth factors were downregulated following time spent in culture. In particular, IGF1 and 2 were found to be significantly (> 20-fold) downregulated in the microarray dataset between day 0 and day 28. However, we also wanted to assess the expression levels of other key factors reported to be important to ASC paracrine function that may be differentially expressed in conditioned media taken from MACS-derived ASC compared to culture-derived ASC. These included IFNg and IL-8 where no changes in mRNA were observed at any time point in the microarray data, and IL-6, VEGF and TGFb 2 where a modest twofold–threefold downregulation was observed between day 0 and day 3 (data not shown). Milliplex^®^ multiplexed assay panels were employed to compare the expression levels of VEGF, IL-6, IL-8, IFNg, TGFb 1 and 2 and IGF-1 and 2 in conditioned media harvested either at day 4 or at day 7 of culture (day 7 representing conditioned media from day 4 to day 7). At day 4 significantly higher levels of IL-8, TGFb1 and 2 were secreted from the MACS-derived ASCs compared to the culture-derived ASCs, which continued to day 7 for TGFb2 (**Figure 4**). At the day 7 time point, MACS-derived ASCs secreted significantly more (> 13-fold) IGF1 than culture-derived ASCs. No significant differences were detected for IGF2, IFNg, IL-6 or VEGF.

**Figure 5:**
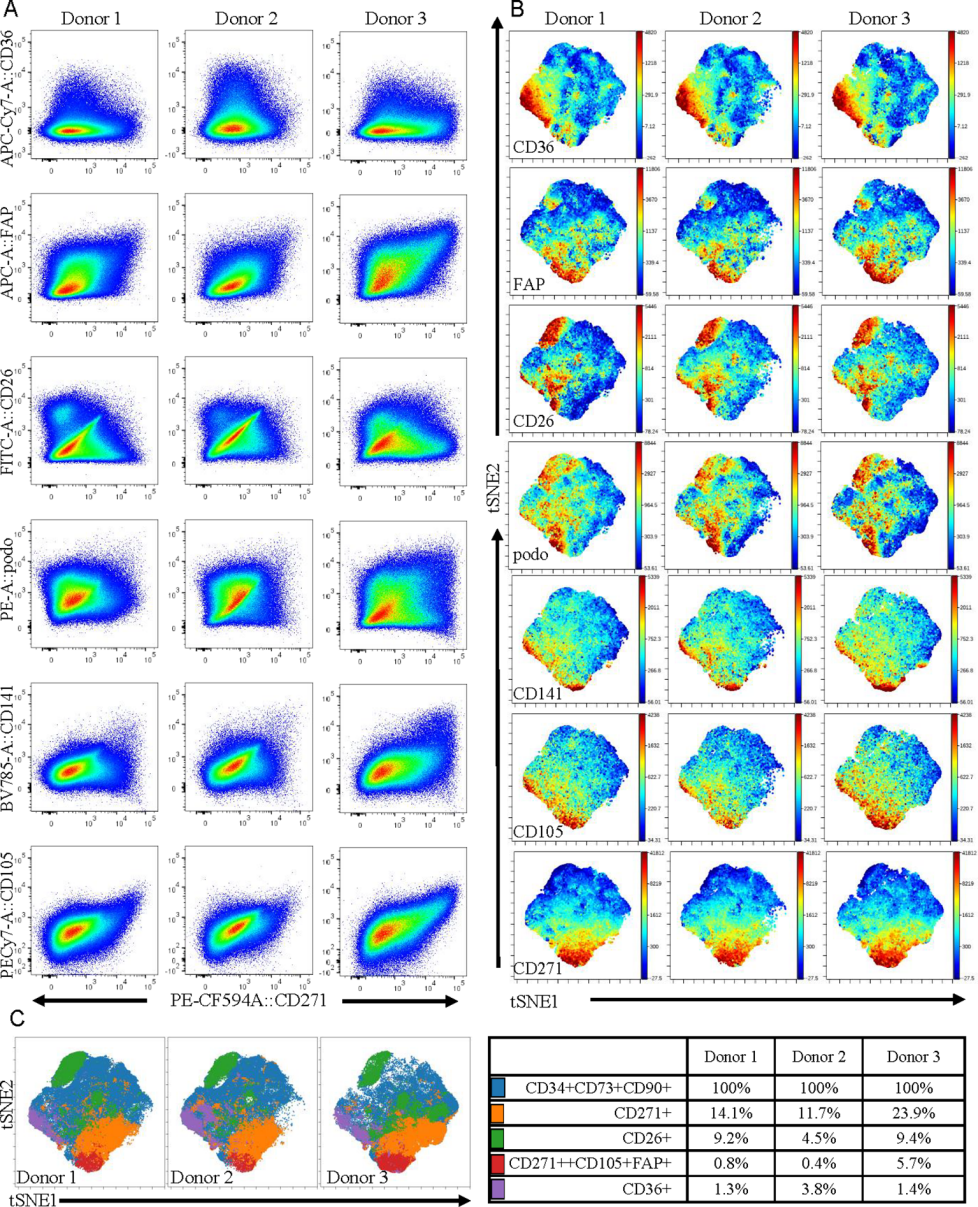
Multicolor flow cytometry of stromal vascular fraction (SVF) indicates that adipose-derived stem cell (ASC) defined by cell-surface expression of CD34, CD73, and CD90 represent a heterogeneous population of cells. **(A)** Multicolor flow cytometry analyses of the SVF using a 16 color flow cytometry indicates that the ASC population is heterogeneous. Following gating exclusion (CD45-CD31-CD146- see **Supplementary Figure 1**), cells with the ASC phenotype, (CD90+CD73+CD34+), identified within SVF were found to be heterogeneous when CD271 was plotted against CD26, CD141, CD36, podoplanin, CD105, and FAPα as labeled, and to differ between donors. Data is derived from three donors. Advanced data analyses were subsequently performed using viSNE **(B)** and FlowSOM **(C)**. viSNE was run using equal sampling (204,977 cells) of the pregated CD90+CD73+CD34+ population per donor to identify subset heterogeneity **(B)**. FlowSOM analyses identified clusters based on expression of podoplanin, CD26, FAPα, CD36, CD141 and CD271 and these are displayed as overlays on the viSNE plots, and percentages of cells present within clusters are tabulated **(C)**.

As the adipose stromal field develops it is becoming increasingly likely that subpopulations of cells exist within the ASC population (Merrick et al., 2019; Raajendiran et al., 2019). Therefore, the observed differences in the expression levels of some key proteins secreted from MACS-derived ASC in comparison to culture-derived ASCs may reflect the fact that these populations have a different cellular composition, even though both populations express the classical ASC markers. To investigate this further we expanded our 11-color flow cytometry panel (Feisst et al., 2014) up to 16 colors to enable a deeper interrogation of the cell surface phenotype of ASC present in uncultured SVF (i.e. the population equivalent to MACS-derived ASC). As we previously reported, ASC constitute a large proportion of the SVF and were positive for CD34, CD73, CD90, and lacked expression of CD31, CD146, and CD45. These CD90+CD73+CD34+ ASC, that are often referred to as homogenous, were analyzed for expression of CD141, FAPa, CD26 (also known as DPP4), CD36, CD271, and podoplanin. HLA-DR, CD144, CD31, and CD146 were also assessed, however expression of these markers was mostly confined to the endothelial and pericytic cell populations rather than the ASCs. Despite the use of a number of new markers, distinct, separable subpopulations were not overly obvious within the ASC population. However, when comparing all markers of interest against CD271, heterogeneity became more apparent (**Figure 5A**). For example, a distinct CD26 positive population was identified that was negative for CD271, suggesting these two markers to be mutually exclusive. Whereas CD271 displayed coexpression with CD105, FAPα, and CD141 across all donors, albeit at different frequencies. Interestingly, the most diverse expression occurred between CD271 and podoplanin and was also highly variable between donors. CD36, which was highly expressed on endothelial populations (data not shown), was also found to be expressed by a subpopulation of ACS. To consider all markers concurrently, we further analyzed this same data set using the advanced analysis algorithms viSNE (**Figure 5B**) and FlowSOM (**Figure 5C**). These analyses reveal the heterogeneity that exists within the CD90+CD73+CD34+ population for the markers podoplanain, CD26, FAPα, CD36, CD141, and CD271. **Figure 5B** demonstrates both the similarities and differences of each of the markers between donors while **Figure 5C** highlights the key populations identified by FlowSOM clustering and the relative differences observed between the three donors. These FlowSOM plots also demonstrate that a proportion of ASC (shown in blue) do not express the markers under investigation. Therefore, our novel 16 color flow cytometry panel reveals that heterogeneity exists not only within the ASC population, but it is also variable between donors. Future studies will therefore be required to further refine the phenotype and determine whether any of these phenotypic characteristics are also functionally relevant.

## Discussion

We present a simple method to enrich an untouched population of defined *ex vivo* ASC from SVF with purity (based on CD34, CD73, and CD90 expression) comparable to ASC isolated using the standard 2–4 weeks in tissue culture method (Feisst et al., 2014). Other groups have reported using an immunomagnetic approach to positively sort ASC from SVF using a single antibodies targeting, for example, CD34 (Maumus et al., 2011), SSEA4 (Jiang et al., 2010; Maddox et al., 2012), CD105 (Jiang et al., 2010), CD49a, CD90, CD-105a, or CD271 (Griesche et al., 2010). Positive sorting using a single antibody will copurify other cell types that also express that marker, and to date no marker has been identified that is exclusively expressed on ASC. In addition, positively selected cells will retain bead-bound antibodies, conferring an advantage to taking a negative selection approach. As far as we are aware we report here on the most comprehensive antibody cocktail that has been employed to negatively enrich for untouched human ASC. Our data indicate that a greater proportion of MACS-derived *ex vivo* ASC exhibit activity in *in vitro* differentiation compared to culture-derived ASC. Increased passage number has previously been reported to result in decreased differentiation potential (Mitchell et al., 2006; Wall et al., 2007; Park et al., 2011; Lo Surdo et al., 2013). Our data extends on these previous results by including an *ex vivo*, uncultured, enriched population of ASC.

The isolation of MACS-derived ASC allowed us to examine the molecular changes that occur within a defined population of cells when subjected to tissue culture. At the RNA level, as expected, we observed no significant change in the expression of stromal cell surface markers CD44, CD73, or CD90 on cultured *ex vivo* MACS-derived cells. We note a previous report showing increased expression of these stromal markers in culture-derived ASC over time until passage 3 (Mitchell et al., 2006), suggesting that at least 3 passages (or approximately 20 days in standard tissue culture conditions) are required to achieve a stromal cell phenotype similar to MACS-derived ASC based on these markers. However, upon culture we did observe a significant downregulation in the expression of stem-cell associated markers CD34 (> 37-fold decrease by day 28) and aldehyde dehydrogenase (ALDH, a 29-fold decrease in RNA levels by day 28). Down regulation of CD34 with time in culture has been reported previously (Mitchell et al., 2006; Yu et al., 2010; Feisst et al., 2014). However, the downregulation of ALDH is a novel observation. We note that a recent paper reported that FACS sorted ALDH-bright ASC were “more primitive” i.e. less differentiated, than their ALDH dim counterparts based on network connectivity parameters using single cell RNA-seq data (Hardy et al., 2017). In addition, we observed a culture-associated reduction in the expression of c-Myc and KLF4 which are genes associated with proliferation and differentiation (Paula et al., 2015; Kami et al., 2016). DAVID analysis of gene expression changes between day 3 and day 28 identified proliferation as a decreased pathway and apoptosis as an increased pathway. This is in line with a report that found a decrease in ASC proliferation rate with time in culture (Legzdina et al., 2016). Expression of some key chemokines reported to be involved in ASC differentiation, migration and wound healing, including CXCL14, CXCL12, CXCL3, and CXCL2 were also significantly downregulated with time in culture (Heneidi et al., 2013; Hayashi et al., 2015; Stuermer et al., 2015; Kusuyama et al., 2016). Collectively the rapid culture-induced changes in gene expression suggest that even a limited time in culture is likely to have a significant impact on ASC activity.

Further marked differences in the profiles of secreted key cytokines were observed when conditioned medium from *ex vivo* MACS-derived ASC and cultured-derived ASC populations were compared. Of the eight proteins we assessed, three were expressed at significantly higher levels in day four conditioned media from MACS-derived ASC compared to culture-derived ASC. These include TGFb1 and 2, which are pleiotropic cytokines with roles in differentiation (Wang et al., 2012) and wound healing (Jung et al., 2011) and IL-8 which is pro-inflammatory and promotes wound healing (MacLeod and Mansbridge, 2016). Levels of IGF1 and 2, which are thought to be antiapoptotic and to have roles in differentiation (Youssef et al., 2017), were on average secreted at greater levels from MACS-derived ASC. However, variability between donors meant that this did not achieve significance except for IFG1 at day 7. Conversely, VEGF, a protein involved in angiogenesis (Johnson and Wilgus, 2014), was secreted on average at greater levels from culture-derived ASC, although again variability between donors meant that this did not achieve significance. The different profiles of secreted proteins from these phenotypically similar populations of cells (based on standard markers of ASC cell surface phenotype) suggest that they may not be functionally similar. They are likely to have different paracrine effects and potentially different clinical utilities. Certainly in settings where high levels of IGF1 secretion are desired, such as in the treatment of myocardial infarction (Bagno et al., 2016), the use of MACS-derived ASC may be more beneficial compared to cultured-derived ASC.

The RNA data generally correlated with the protein data with a notable exception being IL-8. No change was observed at the mRNA level for IL-8 (comparing uncultured MACS-derived ASC to cultured MACS-derived cells) but significant changes were observed in the levels of secreted IL-8 protein in an experiment that compared MACS-derived ASC to cultured-derived ASC. It is possible that, in addition to culture-induced changes, differences in the cellular composition of these two populations may contribute to their different paracrine profiles and activity in differentiation assays. The latter option is feasible as heterogeneity in early passage cultured ASC has been reported previously (Baer et al., 2013; Walmsley et al., 2015; Barilani et al., 2018). Our analysis extends these previous results by showing that heterogeneity and donor-variability exists within uncultured ASC and aligns with recent reports of heterogeneity within this cell population (Merrick et al., 2019; Raajendiran et al., 2019).

Conducting flow cytometry analyses of ASC within uncultured SVF using a novel 16 color flow cytometry panel demonstrated that subpopulations of cells with different expression profiles for the markers CD26, CD36, CD271, CD141, FAPα, and podoplanin exist. Interestingly, the most prominent subpopulation identified within the ASC was positive for CD26 but did not coexpress CD271, a marker that is often described as a key marker on mesenchymal stromal cells (Barilani et al., 2018; Kohli et al., 2019). CD26 has recently been reported to mark highly proliferative, multipotent progenitors present in adipose tissue (Merrick et al., 2019). In the study by Merrick et al. (2019) the CD26+ progenitors were shown to give rise to two distinct types of preadipocytes in the adipose niche. However, the relevance of these newly defined progenitors in terms of their multipotency beyond adipogenesis (i.e. down the osteogenic and chondrogenic mesenchymal lineages) or indeed their therapeutic potential, remains to be explored. CD271 expression has previously been associated with enhanced activity in ASC differentiation assays (Quirici et al., 2010; Barilani et al., 2018; Kohli et al., 2019) and CD36 has been associated with enhanced adipogenic potential (Gao et al., 2017). However, to our knowledge expression of podoplanin, a marker that is used to define lymphatic endothelial cells, has not been reported previously on human uncultured, *ex vivo* ASC. Podoplanin has been found to be expressed on a progenitor population in the liver (Eckert et al., 2016) and has recently been found to regulate mammary stem cell function in mice (Bresson et al., 2018). Therefore, collectively these markers, combined with ALDH as identified in our microarray experiments, may help to define functional subsets within the ASC fraction.

Cell surface marker expression has been reported to vary with time in culture and with different culture conditions (including CD34, CD105 and CD271 (Braun et al., 2013; Barilani et al., 2018)). Therefore, studies such as ours using fresh and uncultured ASC are warranted. CD26, for example, is reported to be broadly expressed in cultured ASC (Baer et al., 2013; Walmsley et al., 2015) whereas we observe a large negative fraction in uncultured cells. Cryopreservation can also affect cell-surface marker expression (Irioda et al., 2016) and based on the above it is likely that some of these markers will be relevant to identifying functional subsets of ASC. Therefore, there is a pressing need for these deeper analyses of *ex vivo* adipose tissue derived mesenchymal stromal cells. Dissecting cellular heterogeneity may lead to a better understanding and, more importantly, the identification of therapeutically relevant cell populations. Indeed, understanding this heterogeneity could enhance the clinical utility of ASC, both as an *ex vivo* or culture-derived cellular product, as well as be informative to understanding their role in the adipose niche.

## Data Availability Statement

The datasets generated for this study can be found in the GEO using accession number GSE136633.

## Ethics Statement

The studies involving human participants were reviewed and approved by the Northern A Health and Disability Ethics Committee. The patients/participants provided their written informed consent to participate in this study.

## Author Contributions

HS and AB conceived and obtained funding for the study. HS, VaF, and AB designed the experiments and analyzed the results. MI, EW, TD, and VJ-P executed most of the experiments. JJ provided technical expertise. RD provided guidance, expertise and obtained funding. ViF assisted with data analysis. HS and AB wrote the manuscript.

## Funding

Arthritis NZ Research Grant: $76,000 technician salary and consumables grant—A clinically relevant protocol to rapidly isolate maximum potency Adipose-Derived Stem Cells from adipose tissue 2016.

Maurice Wilkins Centre—Optimisation of a multicolour flow cytometry panel to characterise mesenchymal cell subsets in normal and malignant human tissue 2015—$10,000 consumables grant.

Maurice Wilkins Centre—Characterisation of adipose tissue progenitor cells: in search of the beige/brite fat precursor cells 2017—$10,000 consumables grant.

Maurice Wilkins Centre—Defining the adipocyte pre-cursor and stem cell populations in human adipose tissue using single-cell RNAseq 2017—$10,000 consumables grant.

University of Auckland FRDF new staff grant: $30,000 consumables grant ‘Investigation into the Molecular Mechanisms of Adipose-Derived Stem Cell Differentiation Potential” 2013.

University of Auckland PBRF Discretionary Fund recipient: $5,000 consumables grant “A clinically relevant protocol to rapidly isolate potent Adipose-Derived Stem Cells from adipose tissue” 2015.

Maurice Wilkins Centre - Principal Investigator funding to RD.

## Conflict of Interest

The authors declare that the research was conducted in the absence of any commercial or financial relationships that could be construed as a potential conflict of interest.
