# Supplementary material for: Ex Vivo Human Adipose Tissue Derived Mesenchymal Stromal Cells (ASC) Are a Heterogeneous Population That Demonstrate Rapid Culture-Induced Changes
Source: Front Pharmacol. 2020 Feb 20;10:1695. doi: 10.3389/fphar.2019.01695 (PMC7044177; doi:10.3389/fphar.2019.01695)
Supplement: Supplementary file 1 [file Table_1.docx]

Supplementary Table S1: BD SORP FACS AriaII with the listed optical elements.

| Laser wavelength | Laser power | Detectors | Band Pass | Spectral Range for Detector (nm) | Dichroic Filter | Fluorophores |
| --- | --- | --- | --- | --- | --- | --- |
| Blue 488nm | 100mw | E | 530/30 | 515-545 | 505LP | Alexa-488/FITC |
|  |  | D | 575/25 | 562.5-587.5 | 550LP | PE |
|  |  | C | 610/20 | 600-620 | 600LP | PE-CF594 |
|  |  | B | 695/40 | 675-715 | 635LP | PerCPCy5.5/PE-Cy5 |
|  |  | A | 780/60 | 750-810 | 755LP | PE-Cy7 |
| Red 640nm | 40mw | C | 660/20 | 650-670 |  | APC/Alexa-647 |
|  |  | B | 710/50 | 685-735 | 685LP | Alexa-700 |
|  |  | A | 780/60 | 750-810 | 755LP | APC-Cy7 |
| Ultraviolet 355nm | 60mw | C | 820/60 | 790-850 | 770LP | BUV805 |
|  |  | B | 450/50 | 425-475 | 410LP | DAPI |
|  |  | A | 379/28 | 365-393 |  | BUV395 |
| Violet 405nm | 100mw | F | 450/50 | 425-475 |  | BV421 |
|  |  | E | 510/50 | 485-535 | 502LP | V500/BV510/BV480 |
|  |  | D | 610/20 | 600-620 | 600LP | BV605 |
|  |  | C | 660/20 | 650-670 | 630LP | BV650 |
|  |  | B | 710/50 | 685-735 | 685LP | BV711 |
|  |  | A | 780/60 | 750-810 | 750LP | BV785/786 |
