# Supplementary material for: Ex Vivo Human Adipose Tissue Derived Mesenchymal Stromal Cells (ASC) Are a Heterogeneous Population That Demonstrate Rapid Culture-Induced Changes
Source: Front Pharmacol. 2020 Feb 20;10:1695. doi: 10.3389/fphar.2019.01695 (PMC7044177; doi:10.3389/fphar.2019.01695)
Supplement: Supplementary file 2 [file Table_2.docx]

**Supplementary Table S2 : Gating strategy to identify ASC within SVF**

ASC in SVF were identified by the following gating strategy. Cells were first gated by FSC-A and SCC-A followed by doublet exclusion using SSCA/SSC-H. Dead cells were excluded using DAPI. Next hematopoetic (CD45) and endothelial (CD31) cells were excluded before identifying the ASC population as CD73+CD90+CD34+ and CD146-

List of antibody reagents:

| **Antigen** | **Fluorophore** | **Clone** | **Vendor** | **Panel use** |
| --- | --- | --- | --- | --- |
| CD26 | FITC | BA5b | BioLegend | 16 colour |
| Podoplanin | PE | NC-08 | BioLegend | 16 colour |
| CD271 | PECF594 | C40-1457 | BD Biosciences | 16 colour |
| CD144 | PerCpCy5.5 | 55-7H1 | BD Biosciences | 16 colour |
| CD105 | PE-Cy7 | SN6 | ebioscience | 16 colour |
| CD90 | Alexa-700 | 5E10 | BioLegend | MACS purity check /16 colour |
| CD36 | APC-Cy7 | 5-271 | BioLegend | 16 colour |
| FAP | APC | 427819 | R&D | 16 colour |
| CD34 | BUV395 | 581 | BD Biosciences | 16 colour |
| Dead cells | DAPI |  | Life Technologies | MACS purity check /16 colour |
| CD73 | BV421 | AD2 | BioLegend | 16 colour |
| CD31 | BV480 | WM59 | BD Biosciences | 16 colour |
| HLADR | BV605 | G46-6 | BD Biosciences | 16 colour |
| CD45 | BV650 | HI30 | BioLegend | 16 colour |
| CD146 | BV711 | P1H12 | BD Biosciences | MACS purity check /16 colour |
| CD141 | BV785 | M80 | BioLegend | 16 colour |
| CD73 | PE | AD2 | BD Biosciences | MACS purity check |
| CD31 | APC-Cy7 | WM59 | BioLegend | MACS purity check |
| CD45 | BUV395 | clone H130 | BD Biosciences | MACS purity check |
| CD34 | BV510 | 581 | BioLegend | MACS purity check |
