# Supplementary figures and images for: Ex Vivo Human Adipose Tissue Derived Mesenchymal Stromal Cells (ASC) Are a Heterogeneous Population That Demonstrate Rapid Culture-Induced Changes
Source: Front Pharmacol. 2020 Feb 20;10:1695. doi: 10.3389/fphar.2019.01695 (PMC7044177; doi:10.3389/fphar.2019.01695)

**Supplementary Figure S1: Gating strategy, antibody reagents and use**

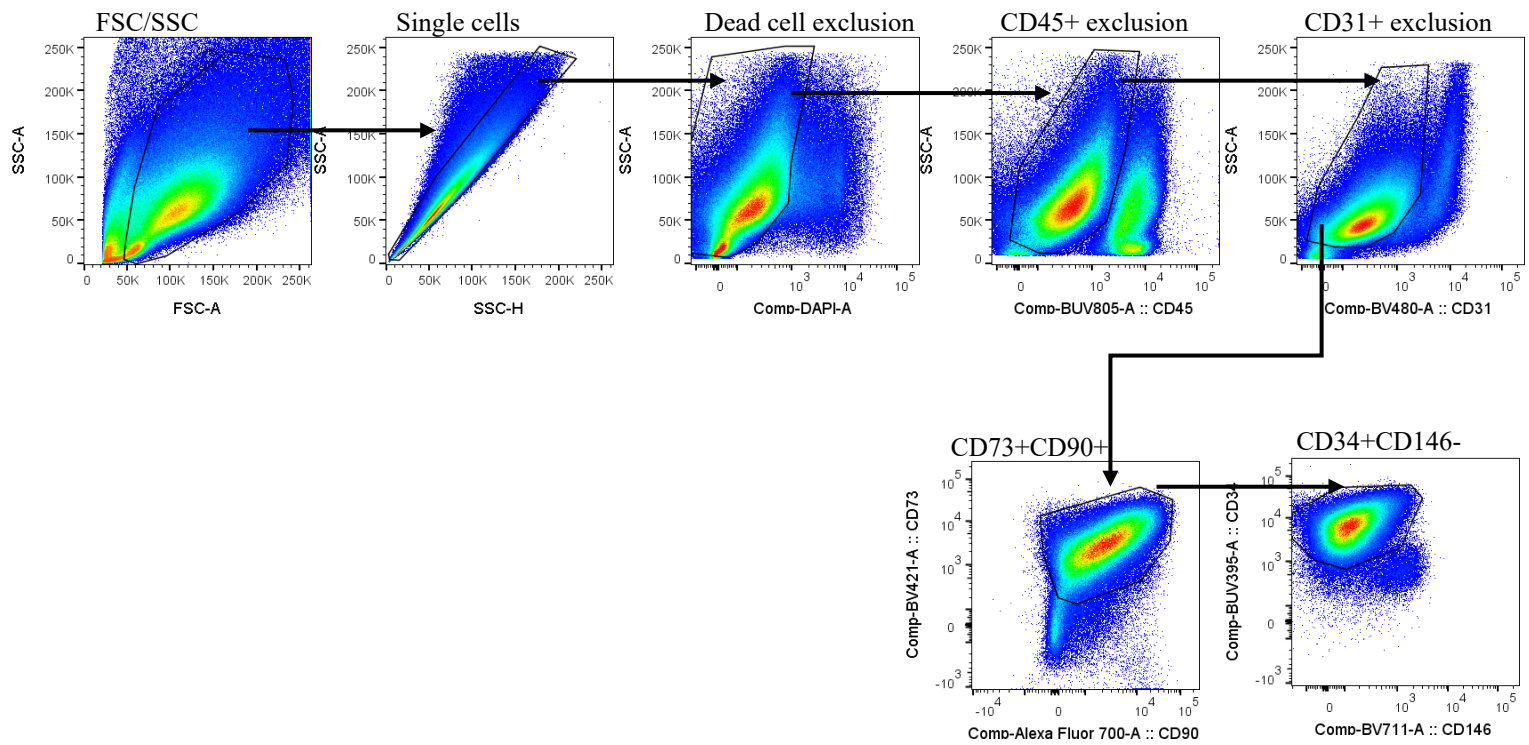

Supplement: Supplementary file 4 [file Image_1.pdf]
